# Magistral Galenic Preparations in Modern Dermatology: Our Top 10 Picks for Bridging Therapeutic Gaps

**DOI:** 10.3390/medicina62030559

**Published:** 2026-03-17

**Authors:** Edoardo Cammarata, Elia Esposto, Laura Cristina Gironi, Elisa Zavattaro, Paola Savoia

**Affiliations:** 1 Struttura Complessa a Direzione Universitaria Dermatologia e Venereologia, Azienda Ospedaliero-Universitaria Maggiore della Carità di Novara, 28100 Novara, Italy; elia.esposto@maggioreosp.novara.it (E.E.); lcristina.gironi@maggioreosp.novara.it (L.C.G.); 2 Department of Health Science, University of Eastern Piedmont, 28100 Novara, Italy; elisa.zavattaro@med.uniupo.it

**Keywords:** magistral preparations, galenic formulations, personalized dermatology

## Abstract

*Background/Objectives:* Topical treatment efficacy is fundamentally dependent on effective delivery of the active pharmaceutical ingredient and its compatibility with the compromised skin barrier. Many commercially available industrial formulations contain poorly tolerated excipients or lack essential therapeutic combinations, frequently leading to complex polypharmacy and reduced patient adherence. In contrast, magistral galenic preparations offer a degree of therapeutic personalization unmatched by standardized products, positioning the compounding laboratory as a strategic resource in dermatological care. This analysis aims to identify and evaluate ten indispensable magistral formulations selected based on their high clinical frequency and the absence of equivalent, globally available commercial alternatives. *Materials and Methods:* Each formulation was according to four strategic pillars: (i) dosage customization, (ii) excipient modification (removing allergens like parabens or fragrances), (iii) synergistic ingredient association, and (iv) vehicle optimization. The dermatological conditions addressed include pediatric scabies, melasma, hidradenitis suppurativa, and autoimmune mucosal diseases. Key selections include Kligman’s formula for hyperpigmentation and personalized trichological preparations. *Results:* The identified “top 10” magistral formulation reveals significant gaps within the standardized pharmaceutical market. In pediatric scabies (specifically patients < 15 kg), benzyl benzoate and precipitated sulfur demonstrate superior efficacy over permethrin, addressing emerging resistance patterns. For acute inflammatory dermatoses, Hoffmann Paste and Lime Liniment provide effective protective barriers while neutralizing local acidity. Antiseptic and astringent solutions, including Burow’s and Silver Nitrate (AgNO_3_) offer targeted mechanisms and biocidal activity, often absent in standardized topicals. Furthermore, specialized adhesive oral pastes for autoimmune conditions minimizing systemic absorption and associated risks. *Conclusions:* Magistral compounding represents a cornerstone of precision medicine in dermatology enabling tailored therapies that bridge critical gaps left by standardized formulations, particularly in complex cases and vulnerable populations.

## 1. Introduction

In dermatology, topical therapy represents the first line of intervention for most skin pathologies. The effectiveness of such interventions depends not only on the active pharmaceutical ingredient (API) but also on its formulation, delivery system and compatibility with a frequently compromised skin barrier [1,2]. Commercially available preparations while standardized and widely accessible, may not adequately meet individual patient needs. Therapeutic gaps may arise from several circumstances: (i) unavailability of specific active ingredients or concentrations in certain countries; (ii) absence of suitable formulations for pediatric, mucosal, or anatomically sensitive areas; (iii) intolerance to industrial excipients such as fragrances, lanolin, parabens, or propylene glycol; and (iv) the lack of commercially available combinations required for complex dermatologic conditions. Galenic preparations, particularly magistral formulations—compounded according to an individual patient’s prescription—provide a level of therapeutic personalization unmatched by standardized industrial products. As such, the compounding laboratory represents an indispensable strategic resource for the management complex and sensitive dermatological conditions [3,4]. This advantage derives from four core capabilities: (i), Dosage Customization, which is essential in pediatric patients or when treating extensive or delicate anatomical areas, where the active pharmaceutical ingredient must be titrated to specific levels unavailable in marketed products; (ii), Excipient Modification, arguably the most critical advantage, as it enables the removal of common allergens or irritants -such as parabens, fragrances, lanolin, or propylene glycol- to which patients with atopic dermatitis or eczema may be sensitized, thereby improving tolerability and treatment adherence; (iii), Synergistic Association, which provides the ability to combine multiple active ingredients within a single vehicle, streamlining the therapeutic regimen, reducing topical polypharmacy, and enhancing their synergistic effect; and (iv), Selection of the Appropriate Vehicle, whereby the choice of an optimal base (liquid, semisolid, or solid) directly influence drug release, penetration, occlusion, and ultimately shaping therapeutic performance. It is critical to recognize that the vehicle in a compounded formulation is not merely an inert carrier of the active pharmaceutical ingredient; rather, it often possesses intrinsic pharmacological properties, that can significantly influence the overall therapeutic outcome, including drug absorption, tolerability, and local effects [5,6].

The safety of magistral preparations depends on the quality of ingredients, strict adherence to good compounding practices, and appropriate storage, with particular emphasis on maintaining sterility for injectable and ophthalmic formulations. Although regulatory requirements differ between countries, pharmacists are ultimately responsible for ensuring product quality, accurate labeling, and patient safety.

The aim of this analysis is to provide a structured clinical framework for ten high-frequency magistral dermatologic formulations selected on the basis of defined therapeutic gaps, clinical relevance, and feasibility of safe compounding, together with eligibility criteria and practical safeguards for their appropriate use.

## 2. Clinically Driven Selection of Essential Magistral Formulations

The ten specific magistral compounding formulations detailed in the subsequent templates were selected based on (i) high frequency of use in routine dermatologic practice; (ii) the presence of a clearly identifiable therapeutic gap, defined as the absence of equivalent commercially available formulations, unsuitable fixed concentrations, excipient intolerance, or regional regulatory unavailability; (iii) an acceptable safety profile when prepared under recognized compounding standards; and (iv) practical feasibility of preparation with commonly accessible pharmaceutical-grade ingredients.

This structured approach was intentionally adopted to avoid compiling an exhaustive but impractical list of potential compounded combinations, instead prioritizing preparations considered therapeutically indispensable and supported by clinical rationale. For each selected formulation, standardized preparation parameters and safety considerations are detailed to enhance reproducibility and quality assurance. The templates also include examples of standard of care for the same conditions; however, these may vary depending on the legislation and clinical guidelines of each country [7,8,9,10,11,12,13,14,15,16]. Table 1 summarizes a practical Vehicle Decision Matrix for magistral preparations, designed to guide clinicians in selecting the most appropriate base according to lesion morphology, anatomical site, patient age, skin sensitivity, and desired pharmacokinetic profile. 

Table 2, Table 3, Table 4, Table 5, Table 6, Table 7, Table 8, Table 9, Table 10 and Table 11 summarized our “top 10” magistral formulation.

The therapeutic mechanisms of these three galenic preparations are fundamentally distinct. Potassium permanganate acts as a powerful, non-specific oxidizing agent, primarily utilized for its disinfectant and drying properties in acute, weeping dermatoses. In contrast, Burow’s solution leverages its astringent properties and acidic pH to exert a mild bacteriostatic effect, focusing clinically on the physical management of edema and exudate rather than primary microbial eradication. Finally, silver nitrate relies on the biocidal activity of the heavy metal cation (Ag+) to achieve potent bactericidal effects, rendering it indispensable for the management of severely infected wounds and burns.

## 3. Discussion and Conclusions

Magistral galenic preparations are far more than mere alternatives to commercial drugs; they constitute a cornerstone of precision medicine in dermatology. By enabling the customization of dosage, excipients, synergistic ingredient combinations, and vehicle selection, these formulations allow clinicians to “design” treatments that are precisely aligned with individual patient needs. This tailored approach not only optimizes the therapeutic efficacy of active pharmaceutical ingredients but also enhances patients’ safety, tolerability and adherence by eliminating poorly tolerated or allergenic excipients commonly present in standardized products. Importantly, in the current therapeutic landscape, magistral compounding should not be viewed as a competitor to industrial pharmaceutical production, but rather as a complementary and strategically distinct approach. While industry provides large-scale, standardized, stability-tested formulations, compounding retains the unique capacity to address therapeutic gaps, rare indications, excipient intolerance, and the need for individualized dosing or vehicle adaptation. In this evolving environment, the future of dermatologic compounding may increasingly lie in high-complexity, precision-oriented niches, where personalization and flexibility are essential and commercially available products are insufficient [17].

The strategic integration of magistral compounding, exemplified by our “top 10” clinical selections, provides a practical framework for identifying formulations that address the most pressing therapeutic gaps. These formulations are particularly valuable in complex dermatological cases such as pediatric populationz, patients with extensive or sensitive lesions or those with rare or refractory conditions. At the same time, the boundary between therapeutic galenic preparations and advanced dermocosmetic science is progressively narrowing. Emerging vehicle technologies—including liposomal and nanoemulsion-based systems, polymeric film-forming bases, and barrier-repair-oriented formulations—offer opportunities to enhance drug penetration, cosmetic acceptability, and patient adherence [18,19,20,21,22]. The integration of dermocosmetic innovation into magistral practice may represent a significant future direction, particularly for chronic inflammatory and barrier-disrupted conditions, where long-term tolerability is as critical as pharmacologic potency.

Our selection strategy underscores the unique and indispensable role of compounding pharmacy in dermatological clinical practice, bridging therapeutic gaps where standardized, marketed options are insufficient or nonexistent. In this context, magistral formulations are not simply alternatives—they are essential tools that elevate the standard of care, reinforce clinical flexibility, and expand the boundaries of what is achievable in dermatologic therapy, while adapting to a rapidly changing pharmaceutical and technological landscape.

This work has several limitations. First, the “top 10” formulations were selected based on clinical relevance, frequency of use, and unmet need rather than through a formal systematic review or meta-analysis, which may introduce selection bias. Second, stability data for many compounded preparations remain limited in the literature; therefore, beyond-use dates were conservatively aligned with recognized standards (e.g., USP <795>) when product-specific evidence was unavailable. Third, regulatory frameworks and compounding permissions vary across jurisdictions, potentially limiting the universal applicability of some recommendations. Finally, while risk-benefit considerations and safety guardrails are provided, real-world outcomes may differ depending on patient characteristics, compounding quality, and adherence to protocols.

Future research should prioritize standardized stability studies, comparative effectiveness data, and multicenter evaluations to further strengthen the evidence base supporting dermatologic magistral preparations.

## Figures and Tables

**Table 1:** Vehicle Decision Matrix for Magistral Preparations.

Skin/Lesion Type	Recommended Vehicles	Vehicles/ Excipients to Avoid	Notes/Rationale
Weeping/ exudative lesions	Burow’s solution, Potassium permanganate solution, Silver nitrate solution (aqueous)	Petrolatum, anhydrous bases	Water-based vehicles help absorb exudate and allow wet dressings; avoid occlusive anhydrous vehicles that trap moisture and bacteria.
Hyperkeratotic/ thick plaques	Zinc oxide paste, anhydrous sticks, MCT oil, petrolatum	Aqueous gels, low-viscosity lotions	Occlusive and viscous bases enhance penetration and retention of actives; minimize irritation and ensure adequate coverage.
Intertriginous/ moist folds	Low-irritant creams, silicone-based ointments, HEC gels	Fragrances, parabens, lanolin, propylene glycol	Vehicles should be non-occlusive yet protective; avoid allergens and irritants that worsen maceration or inflammation.
Mucosal (oral/perioral)	Hydroxyethyl cellulose (HEC) adhesive gels/pastes	Standard cutaneous creams, strong preservatives, fragrances	Hydrophilic, mucoadhesive bases ensure retention on mucosa; prevent systemic absorption and local irritation.
Pediatric/sensitive skin	Olive oil, anhydrous zinc paste, HEC gel, mild cream bases	Fragrances, parabens, lanolin, strong preservatives	Gentle, non-irritating, preservative-free vehicles reduce risk of allergic reactions and improve adherence.
Photolabile/ oxidation-prone actives (hydroquinone, tretinoin, minoxidil, latanoprost)	Light-protective cream or opaque tube; anhydrous/low-water base	High-water bases without antioxidants, alcohols, strong oxidizers	Vehicle selection preserves chemical stability; store in opaque containers and advise proper handling.

**Table 2:** Human Sarcoptic Scabies.

Preparation Name/Suggested Formula.	- Benzyl benzoate 5–25% in sweet almond oil or base cream - Precipitated sulfur 5–17% in base cream or petroleum jelly
Method of Administration	Apply topically to the entire body (from the neck down; include the head for patients < 2 years) *: - Benzyl benzoate: leave on for 8–12 h (4 h for infants < 2 months) * before washing. - Sulfur: apply for 3 consecutive days (Days 0, 1, 2) without bathing to ensure continuous contact.
Advantages of Compounded Preparation	- Effective alternatives to permethrin. - Dosage customization for vulnerable pediatric populations. - Ability to modify vehicle for excipient sensitivities.
Standard of Care/Commercial Products	- Permethrin 5% cream. - Ivermectin oral tablets. - Benzyl benzoate lotion/emulsion. - Malathion 0.5% lotion/liquid. - Crotamiton lotion or cream.
Target Concentration Range	Benzyl benzoate: 5–25% Sulfur: 5–17%
Recommended Vehicle/Base	Sweet almond oil, neutral base cream, petroleum jelly.
Brief Compounding Steps	- Mix the active ingredient thoroughly into the selected vehicle until homogeneous. - Minimize contamination during handling.
Compatibility Constraints	- Avoid combinations with oxidizing agents that may degrade sulfur. - Protect oil-containing vehicles from direct light to prevent oxidation.
Container/Closure	Opaque tube or jar with airtight closure.
Storage Conditions	Store at room temperature, away from direct light and heat sources
Beyond-Use Date (BUD)	- Aqueous or high-moisture preparations: 14 days at controlled temperature - Anhydrous preparations (petroleum jelly/oil-based): 180 days Reference: USP <795>
Safety Notes/Precautions	- Avoid ocular and mucosal contact. - Monitor exposure time carefully in young children. - Educate caregivers on washing, clothing, and bedding precautions.

* Notes: Caregivers of pediatric patients were provided with clear oral and written instructions emphasizing proper application (including trimming the child’s fingernails, gentle application to dry skin, and avoiding sensitive areas) and essential household hygiene measures to prevent reinfestation.

**Table 3:** Bacterial Infections (Antisepsis/Antibiotic/Astringent).

Preparation Name/Suggested Formula	- Potassium permanganate solution 1:10,000 (0.01%) in water - Burow’s solution (stock preparation): Aluminum sulfate 22.5 g, Calcium carbonate 10 g, Acetic acid 25 mL, Purified water 75 mL; further diluted 1:10 to 1:40 before use - Silver nitrate 0.5% solution in purified water or physiological saline
Method of Administration	Apply topically to exudative ulcers or inflammatory dermatoses via soaking or wet dressings for 10–15 min, 1–3 times daily. Avoid prolonged continuous occlusion unless clinically indicated.
Advantages of Compounded Preparation	- Precise dilution control for safety. - Customizable concentration depending on severity and anatomical site. - Useful alternatives when commercial antiseptics are poorly tolerated. - Adjustable dilution for fragile skin or pediatric use.
Standard of Care/Commercial Products	- Chlorhexidine gluconate 2–4% solution. - Povidone-iodine 10% solution - 70% Isopropyl alcohol. - Hydrogen peroxide 3%. - Silver sulfadiazine cream.
Target Concentration Range	Potassium permanganate: 1:10,000 (0.01%) standard; lower concentrations (e.g., 1:20,000) for sensitive areas. Burow’s solution: typically diluted 1:10 to 1:40 before application. Silver nitrate: 0.5% for antimicrobial effect (lower concentrations for fragile skin).
Recommended Vehicle/Base	Aqueous solution (sterile or purified water as appropriate); saline acceptable for silver nitrate.
Brief Compounding Steps	- Accurately weigh active ingredients using calibrated equipment. - Dissolve fully in purified water with continuous stirring. - For Burow’s solution: prepare stock solution, filter, if necessary, then dilute prior to use. - Label clearly with final concentration and dilution instructions. - Prepare in light-protected conditions for silver nitrate.
Compatibility Constraints	- Potassium permanganate: incompatible with organic matter and reducing agents; may stain skin and fabrics. - Burow’s solution: acidic pH; avoid mixing with alkaline substances. - Silver nitrate: light-sensitive; reacts with chlorides forming precipitates; avoid contact with reducing agents.
Container/Closure	Amber glass bottle with airtight closure (especially for silver nitrate and permanganate); clearly labeled for external use only.
Storage Conditions	Store at controlled room temperature, protected from light. Discard if precipitation, color change, or turbidity occurs.
Beyond-Use Date (BUD)	As aqueous formulations without preservatives: up to 14 days when stored at controlled room temperature (USP <795> guidance for water-containing topical preparations), unless stability data support otherwise. Prepare in small quantities to minimize degradation risk.
Safety Notes/Precautions	- Avoid use on face, genital, or large body surface areas unless specifically indicated. - Risk of chemical burns with excessive concentration or prolonged exposure. - Silver nitrate may cause skin staining and, rarely, argyria with chronic use. - Use protective barriers on peri-wound skin. - Discontinue if severe pain, erosion, or worsening inflammation occurs. - Avoid ocular contact.

Notes: (i) Protect surrounding healthy skin with barriers (petroleum jelly or silicone-based ointments); (ii) Monitor for pain, erythema, or erosion; discontinue if adverse reactions occur; (iii) Follow safe handling practices (gloves, correct dilution) to prevent chemical burns, staining, or argyria (silver salts).

**Table 4:** Cutaneous Leishmaniasis.

Preparation Name/Suggested Formula	Paromomycin sulfate 15% cream (Optional combination: Paromomycin sulfate 15% + Gentamicin sulfate 0.5%) * + Urea 6.75% + Purified Water 42.25% + Cream base q.s. to 50 g
Indication/Lesion Eligibility	Suitable for localized cutaneous leishmaniasis: small, non-facial, non-mucosal lesions; limited number of lesions; absence of systemic involvement. Not recommended for mucosal disease or extensive ulceration without specialist supervision.
Method of Administration	Apply a thin layer once daily for 20 consecutive days. Apply directly to cleaned lesion surface. Occlusion may be used if tolerated but is not mandatory. Avoid application on large, ulcerated surfaces without close monitoring.
Advantages of Compounded Preparation	- Demonstrated superiority over vehicle-control formulations in localized cutaneous leishmniasis. - Avoids systemic toxicity associated with pentavalent antimonials (e.g., cardiotoxicity, nephrotoxicity). - Comparable cure rates for selected localized lesions. - Allows combination therapy (paromomycin ± gentamicin) where clinically justified.
Standard of Care/Commercial or Established Therapies	- Paromomycin 15–20% ointment (where commercially available) - Intralesional antimonials (e.g., sodium stibogluconate, meglumine antimoniate) - Cryotherapy
Target Concentration Range	Paromomycin: 15–20% Gentamicin: 0.5% Urea: 5–10% (keratolytic penetration enhancer)
Recommended Vehicle/Base	Hydrophilic cream base (oil-in-water emulsion) to enhance local penetration and tolerability.
Brief Compounding Steps	- Accurately weigh active ingredients. - Dissolve paromomycin and gentamicin in purified water phase. - Incorporate urea into aqueous phase. - Gradually blend aqueous phase into cream base under continuous mixing until homogeneous. - Avoid excessive heat to prevent degradation.
Compatibility Constraints	- Maintain appropriate pH to preserve aminoglycoside stability. - Avoid mixing with strongly alkaline substances. - Monitor for phase separation in high-water formulations. - Use caution when combining with other topical irritants.
Container/Closure	Opaque tube with airtight closure to minimize contamination and oxidation.
Storage Conditions	Store at controlled room temperature; protect from excessive heat and light. Do not freeze.
Beyond-Use Date (BUD)	As a water-containing topical preparation without validated stability data: up to 14 days at controlled room temperature (USP <795> guidance), unless supported by stability studies. Recommend dispensing in limited quantities to ensure freshness during treatment cycle.
Safety Notes/Precautions	- Monitor for local irritant dermatitis. - Systemic ototoxicity/nephrotoxicity is unlikely with topical use but caution is advised when applied to large, ulcerated areas. - Avoid use during pregnancy unless benefit clearly outweighs risk (limited data). - Discontinue if no clinical improvement after full treatment cycle; consider alternative therapy. - Avoid periocular and mucosal application.

* Notes: Topical gentamicin carries minimal risk of ototoxicity or nephrotoxicity: exercise caution when applying to large or ulcerated lesions. Alternative treatments should be considered in pregnancy.

**Table 5:** Oral Bullous/Erosive Autoimmune Diseases.

Preparation Name/Suggested Formula	Clobetasol propionate 0.05% in oral adhesive paste + Hydroxyethyl cellulose 4% + Adhesive base q.s. to 50 g
Indication	Oral bullous or erosive autoimmune diseases (e.g., erosive oral lichen planus, mucous membrane pemphigoid, pemphigus vulgaris with limited oral involvement).
Method of Administration	Apply a pea-sized amount directly to dried affected mucosa twice daily using a clean finger or applicator. Ensure a dry field before application to optimize adhesion. Avoid eating or drinking for at least 30 min after application. Continue until complete re-epithelialization, then taper to lower-potency therapy for maintenance if needed.
Advantages of Compounded Preparation	- Provides a mucoadhesive, biocompatible vehicle specifically designed for oral mucosa. - Avoids inappropriate use of cutaneous steroid creams intra-orally. - Improves local retention time and therapeutic efficacy. - Reduces systemic absorption risk compared with non-adhesive commercial vehicles.
Standard of Care/Systemic Therapies	- Systemic corticosteroids - Azathioprine - Mycophenolate mofetil - Rituximab - Adjunctive analgesics
Target Concentration Range	Clobetasol propionate: 0.025–0.05% Hydroxyethyl cellulose: 3–5% for optimal mucoadhesion
Recommended Vehicle/Base	Mucoadhesive oral paste based on hydroxyethyl cellulose; non-irritating, alcohol-free, and free of flavoring agents or common sensitizers.
Brief Compounding Steps	- Accurately weigh clobetasol. - Levigate active pharmaceutical ingredients to fine powder for uniform dispersion. - Incorporate into pre-hydrated Hydroxyethyl cellulose gel base under continuous mixing until homogeneous. - Avoid excessive shear that may reduce viscosity. - Ensure final preparation has smooth, uniform consistency.
Compatibility Constraints	- Avoid incorporation into acidic or highly alkaline bases that may affect stability. - Do not combine with antifungal agents in the same base unless compatibility is confirmed. - Avoid alcohol-containing vehicles.
Container/Closure	Opaque collapsible tube with narrow tip to reduce contamination and improve dosing precision.
Storage Conditions	Store at controlled room temperature; protect from excessive heat. Keep tightly closed.
Beyond-Use Date (BUD)	As a water-containing mucoadhesive preparation without validated stability data: up to 14 days at controlled room temperature (USP <795> guidance). Dispense in limited quantities to ensure stability.
Safety Notes/Precautions	- Maximum recommended use: short-term induction therapy; avoid prolonged continuous use without reassessment. - Monitor for oral candidiasis; consider antifungal prophylaxis in high-risk patients. - Advise against swallowing the preparation. - Avoid use on extensive mucosal surfaces without specialist supervision. - Gradual taper to lower-potency topical corticosteroid or tacrolimus for maintenance. - Do not substitute with cutaneous clobetasol creams for intraoral use.

**Table 6:** Diaper Dermatitis, Intertrigo, and Localized Irritation.

Preparation Name/Suggested Formula	Hoffmann Paste Zinc oxide 25–50% in olive oil Optional addition (short-term use only): Betamethasone valerate 0.1% or Mometasone furoate 0.1%
Indication	Diaper dermatitis, intertrigo, localized irritative dermatitis with maceration and erythema. Not indicated for untreated fungal infection unless combined with appropriate antifungal therapy.
Method of Administration	Apply a thin but covering layer to affected area 2–4 times daily for 10–20 days. In diaper dermatitis, apply at each diaper change. If corticosteroid is included, limit use to short courses (typically ≤7–10 days).
Advantages of Compounded Preparation	- Allows adjustment of zinc oxide concentration (25–50%) depending on severity. - Preservative-free option for highly sensitive or atopic patients. - Ability to avoid industrial stabilizers or excipients. - Option to incorporate short-term anti-inflammatory therapy when clinically justified.
Standard of Care/Commercial Products	- Barrier creams (zinc oxide–based) - Clotrimazole (if candidal involvement) - Hydrocortisone 0.5–1% (mild inflammation)
Target Concentration Range	Zinc oxide: 25–50%. Betamethasone valerate: 0.05–0.1%. Mometasone furoate: 0.05–0.1%
Recommended Vehicle/Base	Olive oil–based paste (anhydrous). Ensure high-viscosity consistency to maintain barrier effect.
Brief Compounding Steps	- Accurately weigh zinc oxide and levigate to fine powder. - Gradually incorporate olive oil with geometric dilution to achieve homogeneous dispersion. - If adding corticosteroid, pre-levigate separately before incorporation. - Mix thoroughly to minimize sedimentation.
Compatibility Constraints	- Risk of sedimentation and phase separation (zinc oxide compaction). - Shake or stir before dispensing if separation observed. - Avoid combination with strong acids or oxidizing agents. - Use caution if combining with antifungals unless compatibility confirmed.
Container/Closure	Wide-mouth opaque jar or collapsible tube to allow dispensing of thick paste. Ensure airtight closure.
Storage Conditions	Store at room temperature; protect from excessive heat. Stir before use if oil separation occurs.
Beyond-Use Date (BUD)	As anhydrous topical preparation: up to 90 days (USP <795> guidance for non–water-containing topical formulations), unless stability concerns arise. If corticosteroid is added, consider shorter BUD (e.g., 60 days) in absence of stability data.
Safety Notes/Precautions	- Avoid prolonged corticosteroid use in occluded areas (risk of skin atrophy, systemic absorption, especially in infants). - Limit treated surface area when corticosteroids are included. - Reassess if no improvement within 5–7 days. - Consider fungal superinfection in persistent intertrigo. - Safe for pediatric use when zinc-only formulation is used appropriately.

**Table 7:** Pediatric Care and Soothing Irritated Skin.

Preparation Name/Suggested Formula	Oleo-calcareous liniment (Lime liniment): Limewater (Calcium hydroxide solution) 50% Olive oil 50%
Indication	Diaper area cleansing, cradle cap removal, mild irritant dermatitis, eczema-prone skin, minor superficial burns. Particularly suitable for pediatric and sensitive skin.
Method of Administration	Apply topically 1–2 times daily. May be used as a gentle cleanser for the diaper area (apply with cotton pad, no rinsing required) or as a soothing emollient layer. Shake well before use.
Advantages of Compounded Preparation	- Preservative-free, fragrance-free formulation suitable for infants and sensitive skin. - Customizable oil phase (e.g., olive oil alternatives if needed). - Avoids common industrial irritants and emulsifiers. - Simple, well-tolerated barrier and pH-buffering action.
Standard of Care/Commercial Alternatives	Barrier creams (zinc oxide-based), emollients, ointments for diaper dermatitis.
Target Composition	1:1 ratio (50:50) limewater and olive oil.
Vehicle/Base Type	Biphasic emulsion (aqueous limewater + lipid phase). Water-containing preparation without added preservatives unless specifically formulated.
Brief Compounding Steps	- Prepare or obtain pharmaceutical-grade limewater (calcium hydroxide solution). - Measure equal volumes of limewater and olive oil. - Combine and mix until homogeneous milky emulsion forms. - Dispense in appropriate container with “Shake well before use” label.
Compatibility Constraints	- Avoid addition of acidic substances (may destabilize alkaline component). - Emulsion may separate naturally; shaking restores uniformity. - Microbial stability limited if no preservative added.
Container/Closure	Opaque or light-protected bottle with tight closure; preferably flip-cap to minimize contamination.
Storage Conditions	Store at controlled room temperature. Protect from excessive heat. Discard if odor, color, or phase separation persists after shaking.
Beyond-Use Date (BUD)	Water-containing topical preparation without preservatives: up to 14 days (USP <795> conservative approach). Prepare in small batches.
Safety Notes/Precautions	- External use only. - Avoid direct ocular contact. - Discontinue if irritation develops. - In diaper dermatitis, ensure frequent diaper changes and skin drying. - Protect severely eroded or infected skin; medical evaluation required in such cases.

**Table 8:** Melasma/Hyperpigmentation.

Preparation Name/Suggested Formula	Kligman’s Formula: Hydroquinone 5% + Dexamethasone 0.1% + Tretinoin 0.05–0.1% + Cream base q.s.
Indication	Melasma or localized hyperpigmentation of the skin. Not recommended for widespread or photodamaged skin without dermatologic supervision.
Method of Administration	Apply a thin layer once daily at night. Duration: 2–4 months, with reassessment. Limit continuous use to a maximum of 6 months to reduce risk of ochronosis and steroid-related skin atrophy.
Advantages of Compounded Preparation	- Combines multiple key actives in a single preparation, improving adherence. - Avoids complex topical polypharmacy regimens. - Allows customization of concentrations based on patient tolerance and response. - Useful where commercial combination products are not available.
Standard of Care/Commercial Products	- Hydroquinone 2–4% cream or lotion - Azelaic acid 15–20% - Niacinamide—Tranexamic acid topical formulations
Target Concentration Range	Hydroquinone: 4–5%. Dexamethasone: 0.05–0.1%. Tretinoin: 0.05–0.1%
Recommended Vehicle/Base	Cream base suitable for facial use; non-irritating, light-stable, compatible with hydroquinone and tretinoin.
Brief Compounding Steps	- Levigate hydroquinone powder for uniform dispersion. - Incorporate corticosteroid (dexamethasone) and tretinoin carefully to avoid degradation. - Blend into cream base under low shear. - Protect from light and excessive heat during compounding and storage.
Compatibility Constraints	- Hydroquinone sensitive to light and oxidation. - Tretinoin degrades in high heat or inappropriate pH. - Avoid mixing with strong oxidizers or incompatible acids/bases.
Container/Closure	Opaque tube or jar to protect from light; airtight closure recommended.
Storage Conditions	Store at controlled room temperature; protect from light and heat. Refrigeration optional if local guidelines recommend it for hydroquinone stability.
Beyond-Use Date (BUD)	Water-containing cream: up to 14 days (USP <795>). Anhydrous or low-water base: up to 30 days; prepare in small batches to minimize degradation.
Safety Notes/Precautions	- Monitor for irritation, erythema, or excessive dryness. - Advise sun protection during therapy. - Avoid use on broken or inflamed skin. - Discontinue if ochronosis signs appear. - Limit corticosteroid exposure to facial skin; reassess periodically. - Not recommended during pregnancy or breastfeeding unless clearly indicated.

**Table 9:** Acne/Rosacea.

Preparation Name/Suggested Formula	Dapsone topical gel or cream 2–7.5% Cream or gel base q.s. to desired volume
Indication	Acne vulgaris (inflammatory lesions) or rosacea (papulopustular type). Not for use on extensively broken skin or mucosal surfaces.
Method of Administration	Apply a thin layer once daily to affected areas. Duration: up to 12 weeks, reassess for efficacy and tolerability. Limit maximum treated surface area to avoid systemic absorption.
Advantages of Compounded Preparation	- Provides access to intermediate concentrations and vehicles not always commercially available globally. - Customizable formulation to suit sensitive or rosacea-prone skin. - Facilitates local therapy while minimizing systemic exposure.
Standard of Care/Commercial Alternatives	- Oral antibiotics (tetracyclines) - Topical retinoids (adapalene, tretinoin) - Oral isotretinoin (severe, nodulocystic acne)
Target Concentration Range	Dapsone: 2–7.5% depending on severity and tolerance
Recommended Vehicle/Base	Gel or cream base suitable for sensitive facial skin; non-comedogenic, free of common irritants (fragrances, lanolin, parabens).
Brief Compounding Steps	- Accurately weigh dapsone powder. - Levigate to fine powder to ensure uniform dispersion. - Incorporate gradually into gel or cream base under gentle mixing. - Avoid high heat to prevent degradation.
Compatibility Constraints	- Avoid simultaneous use with strong oxidizing agents; benzoyl peroxide may cause temporary yellow-orange discoloration but is generally safe if patients are counseled. - Avoid highly acidic or alkaline vehicles that may destabilize dapsone.
Container/Closure	Opaque tube or jar with airtight closure to prevent oxidation and contamination.
Storage Conditions	Store at controlled room temperature; protect from light. Refrigeration optional depending on vehicle.
Beyond-Use Date (BUD)	Water-containing topical preparation: up to 14 days (USP <795>). Anhydrous preparation: up to 30 days. Prepare small batches to ensure freshness.
Safety Notes/Precautions	- Rare risk of methemoglobinemia, especially in infants or large body surface application. - Counsel patients about possible temporary yellow-orange staining when used with benzoyl peroxide. - Monitor for irritation or dermatitis in rosacea-prone skin. - Avoid mucosal application. - Discontinue if severe local reactions occur.

**Table 10:** Hidradenitis Suppurativa.

Preparation Name/Suggested Formula	Resorcinol 15% in cream base
Indication	Mild-to-moderate hidradenitis suppurativa with localized lesions, particularly nodules and abscesses. Not recommended for extensive ulceration without specialist supervision.
Method of Administration	Apply a thin layer topically once daily for an initial 6–12-week period. Followed by maintenance therapy (e.g., 2–3 times weekly) based on clinical response. Reassess every 4–6 weeks to adjust frequency and duration.
Advantages of Compounded Preparation	- Provides a topical alternative to clindamycin, particularly in the context of antibiotic resistance. - Allows dose and vehicle customization for sensitive or inflamed areas. - Supports tapering strategy to reduce irritation while maintaining efficacy.
Standard of Care/Commercial Alternatives	- Topical clindamycin 1% - Oral antibiotics (tetracycline, rifampicin/clindamycin) - Systemic biologics (Tumor Necrosis Factor-alpha inhibitors, Inerleuchin-17 inhibitors)
Target Concentration Range	Resorcinol: 15% (standard for acute HS lesions; may adjust lower for sensitive skin)
Recommended Vehicle/Base	Non-irritating cream base suitable for inflamed intertriginous areas; free of fragrances, parabens, and strong preservatives.
Brief Compounding Steps	- Accurately weigh resorcinol. - Levigate to fine powder for uniform dispersion. - Incorporate gradually into cream base under continuous mixing. - Avoid overheating, which can degrade resorcinol.
Compatibility Constraints	- Avoid use with strongly oxidizing or acidic agents. - Avoid combination with topical corticosteroids unless clinically indicated - Do not exceed recommended surface area to minimize local irritation.
Container/Closure	Opaque tube or jar with airtight closure; clearly label “External use only”.
Storage Conditions	Store at controlled room temperature; protect from light and moisture.
Beyond-Use Date (BUD)	Water-containing cream: up to 14 days (USP <795>). Anhydrous cream: up to 30 days; prepare small batches to maintain stability.
Safety Notes/Precautions	- May cause local irritation, erythema, or mild burning. - Limit treated surface area and frequency in sensitive skin areas. - Avoid use in pregnancy and in patients with thyroid disorders unless benefits clearly outweigh risks. - Use a tapering strategy from daily acute therapy to maintenance dosing. - Discontinue if severe irritation or dermatitis occurs. - Do not use on open, infected wounds without medical supervision.

**Table 11:** Androgenic Alopecia.

Preparation Name/Suggested Formula	Combination topical solution/foam/lotion: Minoxidil 5%Finasteride 0.25%Latanoprost 0.02%Vehicle q.s. to desired volume
Indication	Androgenetic alopecia (male and female pattern hair loss). Selected cases of eyelash hypotrichosis (latanoprost component).
Method of Administration	Apply 1 mL once daily to dry scalp, gently massaging into affected areas. For eyelash use: apply a minimal amount along the upper lash line using sterile applicator; avoid ocular instillation.
Advantages of Compounded Preparation	- Combines multiple active agents in a single formulation, improving adherence. - Allows customization of concentrations and vehicles for sensitive scalp. - Enables use of non-irritating, low-propylene glycol vehicles. - Provides access to topical finasteride and latanoprost combinations not universally available commercially.
Standard of Care/Commercial Alternatives	- Topical minoxidil 2–5% - Oral finasteride - Oral antiandrogens (e.g., spironolactone, dutasteride)
Target Concentration Range	Minoxidil 5%Finasteride 0.1–0.25%Latanoprost 0.01–0.02%
Vehicle/Base Type	Hydroalcoholic solution, foam, or lotion. May use low–propylene glycol or propylene glycol–free base for sensitive scalp. Anhydrous or low-water systems preferred for stability.
pH/Solubility Considerations	- Minoxidil requires adequate alcohol/solvent system for solubilization. - Latanoprost is light-sensitive and should be protected from photodegradation. - Avoid extreme pH conditions.
Brief Compounding Steps	- Dissolve minoxidil in appropriate hydroalcoholic solvent system. - Dissolve finasteride and latanoprost in compatible solvent phase. - Combine under controlled mixing until homogeneous. - Protect from light during preparation and dispensing.
Compatibility Constraints	- Avoid strong oxidizers and extreme pH. - Ensure adequate solvent system to prevent minoxidil crystallization. - Latanoprost degrades with light exposure.
Container/Closure	Opaque, light-resistant bottle with pump or dropper. Airtight closure to minimize oxidation and solvent evaporation.
Storage Conditions	Store at controlled room temperature; protect from light and heat.
Beyond-Use Date (BUD)	Hydroalcoholic preparation (non-aqueous or low-water): up to 30 days (USP <795> conservative default). Prepare in small batches due to limited published stability data for combined formulation.
Safety Notes/Precautions	- Avoid use in pregnancy or breastfeeding (finasteride exposure risk). - Monitor for scalp irritation or contact dermatitis. - Counsel regarding possible hypertrichosis outside application area. - Latanoprost: avoid direct ocular exposure. - Reassess efficacy after 3–6 months. - Discontinue if systemic adverse effects occur.

## Data Availability

The original contributions presented in this study are included in the article. Further inquiries can be directed to the corresponding author.
